# Towards environmental friendly multi-step processing of efficient mixed-cation mixed halide perovskite solar cells from chemically bath deposited lead sulphide

**DOI:** 10.1038/s41598-021-97633-5

**Published:** 2021-09-17

**Authors:** Sahel Gozalzadeh, Farzad Nasirpouri, Sang Il Seok

**Affiliations:** 1 Faculty of Materials Engineering, Sahand University of Technology, 51335-1996 Tabriz, Iran; 2 School of Energy and Chemical Engineering, Ulsan National Institute of Science and Technology (UNIST), 50 UNIST-gil, Eonyang-eup, Ulju-gun, Ulsan, 689-798 Republic of Korea

**Keywords:** Materials for energy and catalysis, Solar cells

## Abstract

Organic–inorganic hybrid perovskite is the most promising active layer for new generation of solar cells. Despite of highly efficient perovskite active layer conventionally fabricated by spin coating methods, the need for using toxic solvents like dimethylformamide (DMF) required for dissolving low soluble metal precursors as well as the difficulties for upscaling the process have restricted their practical development. To deal with these shortcomings, in this work, lead sulphide as the lead metal precursor was produced by aqueous chemical bath deposition. Subsequently, PbS films were chemically converted to PbI_2_ and finally to mixed-cation mixed halide perovskite films. The microstructural, optical and solar cell performance of mixed cation mixed halide perovskite films were examined. Results show that controlling the morphology of PbI_2_ platelets achieved from PbS precursor films enabled efficient conversion to final perovskite films. Using this processing technique, smooth and pin hole-free perovskite films having columnar grains of about 800 nm and a bandgap of 1.55 eV were produced. The solar cell performance consisting of such perovskite layers gave rise to a notable power conversion efficiency of 11.35% under standard solar conditions. The proposed processing technique is very promising towards an environmentally friendly method for the production of large-scale high efficient perovskite solar cells.

## Introduction

Organolead halide perovskites with ABX_3_ formula (A: methylammonium (MA), formamidinium (FA); B: Pb, Sn, etc.; X: Cl^−^, I^−^, Br^−^)^1^ are of great interest for solar cell applications owing to their remarkable properties, such as high absorption coefficient^2^, weak electron–hole binding energy^3,4^, adjustable and direct band gap^5^ and long charge carrier diffusion length^6^ and also facile cost-effective fabrication methods. The phenomenal performances of these alluring absorbing layers have skyrocketed their power conversion efficiencies (PCEs) from 3.8^7^ to over 25.2%^8^ in a short period of 10 years for perovskite solar cell (PSC) devices.

Two main routes widely used for synthesizing organometal halide perovskites are single step and sequential deposition methods. For the former one, a solution including both perovskite precursors (PbX_2_ (X: I, Cl, Br) and MAX (MA^+^  = (CH_3_NH_3_)^+^) or FAX (FA^+^  = (CH (NH_2_)_2_)^+^) is deposited via methods like spin coating^9^, spray deposition^10^, blade coating^11^, inkjet printing^12^ and slot die coating^13^ onto a substrate to form the perovskite material. Indeed, the uncontrollable precipitation of the perovskite by a single-step method often results in low reproducibility, morphological variations and a wide spread of photovoltaic performance of the devices^14^. To address these problems, the sequential deposition method was introduced with PbI_2_ layer being deposited from either a solution^14^ phase or a vapor^15^ phase onto substrate firstly and following conversion into perovskite by exposing to a MAX (MA^+^  = (CH_3_NH_3_)^+^) or FAX (FA^+^  = (CH (NH_2_)_2_)^+^) solution or vapor, which better controls the perovskite morphology and its stoichiometry and crystallinity. Therefore, the possibility of photovoltaic enhancement has been shown to be higher with this method^14^ and is anticipated that new routes with further modifications will be required to ever enhance the solar cell performance in this way. For instance, the poor solubility of PbI_2_ and utilization of toxic solvents like dimethylformamide (DMF) makes the first step of the sequential deposition method, i.e., depositing PbI_2_ layer, not well recommended and suitable for upscaling for mass production. One approach for dealing with this problem is to use water-based metal precursor compounds.

There has been a recent trend with respect to the development of such two- or multi-step processing routes for the production of the metal halide perovskite films and solar cells from water-based metal precursors. The general procedure employed is to firstly deposit metal (M) precursor film followed by iodination leading to MI_2_. This finally is chemically converted to MAPbI_3_ perovskite films. In addition, direct conversion of metal precursor to final perovskite has also been reported. A few metal precursors such as Pb^16^, PbO^17,18^, PbO_2_^19,20^, Pb(NO_3_)_2_^21–24^, PbSe^25^ and PbS^26–29^, have been developed via different deposition approaches including chemical, physical and electrochemical methods. Table S1 summarizes different metal precursors, their processing towards the fabrication of PSC and highest PCE of the cell reported.

Lead sulphide (PbS) is a non-halide compound with a lower bond dissociation energy (3.3 eV) compared to oxide precursors^30^ and therefore more chemically reactive which facilitates its subsequent reduction to final perovskite films. PbS can be deposited by various techniques such as vacuum evaporation^31^, spray pyrolysis^32^, successive ionic layer adsorption and reaction^33^, electrodeposition^34^, molecular beam epitaxy^35^, and chemical bath deposition (CBD)^36,37^.

Further to the methods already developed for depositing PbS, some of them have been employed in combination with solution processing to produce final lead halide perovskite film and PSCs. Sutherland et al.^26^ have reported atomic layer deposition of lead sulphide (PbS) layer for the first time. The deposited PbS was a 75 nm thick film introduced as a precursor material to be subsequently converted into the final perovskite layer^26^. Successive exposure to iodine vapor and methylammonium bromide was used to directly convert two-dimensional (2D) PbS nanocrystals into nanocrystals of hybrid perovskite maintaining 2D morphology^38^. Radio-frequency sputtering assisted solution process has also been employed for depositing PbS films which were converted into the perovskite layer by exposing in an iodine atmosphere at room temperature, followed by immersing in a methylammonium iodide solution to be transformed to the perovskite layer^27^. CBD technique combined with chemical vapor deposition (CVD) has been proved to be applicable for synthesizing lead halide perovskite films whose power conversion efficiency has been reported 4.68% in a PSC^28^. We have recently demonstrated that lead halide perovskite films and cells were successfully fabricated based on electrodeposited PbS^29^ with a champion efficiency of 7.72%. The proposed work on the electrodeposition of PbS and subsequent solution based processing towards the fabrication of lead halide perovskite films provided complex interfacial electrode reactions and nucleation and growth with cuboidal grain morphology with uneven interlayer junctions which delays the charge carrier transportation and increases the recombination rates before hitting the hole transport layer.

CBD has been established as a distinct method of depositing PbS with controllable morphology along with its scalability, simplicity, low-cost and low temperature^39–41^. As explained above there is only one report of using CBD for depositing precursor of lead sulphide, which is combined with CVD method for final conversion to lead halide perovskite. MAPbI_3_ has been used as the final product in most of reports. As an alternative precursor material, FAPbI_3_ is shown a potential candidate with its broader absorption spectrum and narrower bandgap material than MAPbI_3_, however, its morphology and phase structure have significant effects on its performance in perovskite solar cells. In most cases, the PCE of FAPbI_3_ based inverted planar devices are lower than those of MAPbI_3_ because of the inferior morphology of FAPbI_3_ film. It is well known that the performance of devices based on MAPbI_3_ would degrade greatly in high temperature or high humidity level. FAPbI_3_ is more stable than MAPbI_3_, but it has an undesirable phase transition between δ-phase (yellow phase; more stable) and perovskite α-phase (black phase) at ambient condition^42^.

It has been shown that adding small amount of MA^+^ induces crystallization of black phase of FA-based perovskite and inhibits the formation of its δ phase^43,44^. A more notable structural stability of the α-phase FAPbI_3_ was achieved by developing a mixed-cation mixed halide perovskite^45^.

Therefore, in this work, we employed a toxic solvent-free route based on CBD to deposit PbS as the lead metal precursor film is introduced for fabricating perovskite films with the mixed cations (FA, MA) and mixed halides (I, Br, Cl). The process started with the chemical bath deposition of a PbS seeding layer. Then, PbS film was exposed to iodine vapor to be chemically converted to PbI_2_. Depending on the temperature of iodination, the morphology of the PbI_2_ layer changed. The effect of different morphologies on final step i.e. chemical conversion to perovskite film as well as photovoltaic performance of achieved layers in the perovskite solar cell were studied. This study represents the versatility of the proposed route to produce a fully covered and high-quality perovskite surface film. After current optimization, the power conversion efficiency (PCE) of 11.35% was achieved under standard conditions (air mass (AM) 1.5, 100 mW cm^−2^) with potential enhancement after several trials in future.

## Results and discussion

### Chemical bath deposition of PbS films

**Figure 1:**
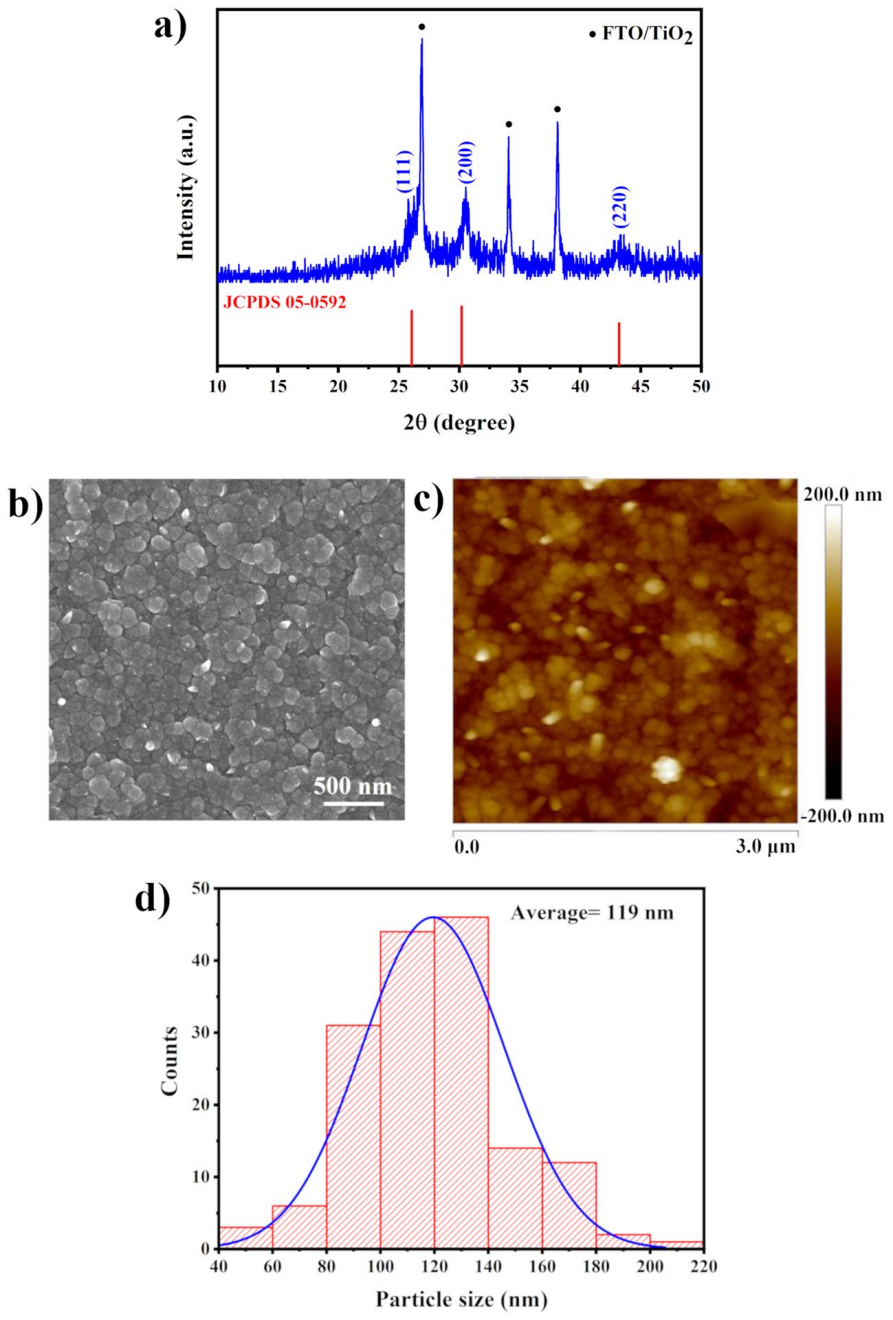
Structural and morphological characterization of chemically deposited PbS film. (**a**) XRD diffraction pattern (**b**) surface SEM image (**c**) AFM image and (**d**) grain size distribution histogram.

Figure 1a shows the XRD pattern of chemically deposited PbS layer. As is evident from the XRD pattern, PbS thin film exhibits a face-centered-cubic (fcc) microstructure. The main Bragg diffraction peaks located at 25.78°, 30.548°, 43.32° correspond to (111), (200) and (220) crystalline planes, respectively (JCPDS No. 00-05-0592). Neglecting the diffraction peaks of PbS and substrate (FTO and TiO_2_), no other peaks were detected which certified the purity of the deposited PbS film. The film formation in the chemical bath deposition method starts with the supersaturation of the solution which results in the production of contents of cations and anions exceeding the solubility. Taking this point into account, the formation of PbS from the aforementioned chemical bath involves the following steps: (1) dissolving Pb(CH_3_COO)_2_ in aqueous solution results in the formation of Pb^2+^ ions, (2) dissociation of thiourea in solution leads to release of SH^−^ ions. SH^−^ ions go through reaction with hydroxide species to produce S^2−^ anions. Finally, PbS thin film forms via adsorbing of Pb^2+^ cations on substrate and combining with S^2−^ anions. The overall growth of PbS film takes place by ion-by-ion process on the substrate. The corresponding reactions are as follows (reactions 1–4)^46^:

1 $$Pb{({CH}_{3}COO)}_{2}\to {Pb}^{2+}+2{{CH}_{3}COO}^{-}$$

2 $${SC({NH}_{2})}_{2}+{OH}^{-}\to {CH}_{2}{N}_{2}+{H}_{2}O+{SH}^{-}$$

3 $${{SH}^{-}+OH}^{-}\to {{S}^{2-}}_{2}+{H}_{2}O$$

4 $${Pb}^{2+}+{S}^{2-}\to PbS$$

SEM micrographs taken from top (Fig. 1b) of the chemically deposited PbS shows a compact structure which consists of smooth, uniform spherical and densely packed grains. The film covers the substrate completely. The thickness of PbS layers deposited for various dipping times as well as the thickness of corresponding PbI_2_ and perovskite films were measured and summarized in Table S2.

Figure 1c represents the AFM image of the PbS thin film deposited on the mp-TiO_2_/bl-TiO_2_/FTO substrate. It indicates that the PbS thin film is composed of particles of granular nature which are smoothly and uniformly distributed on the surface. The root–mean–square roughness (*Rq*) of film is about 31.3 nm. Furthermore, the particle size distribution histogram calculated on the SEM image is shown in Fig. 1d. Accordingly, the average granular grain size of PbS is approximately 119 nm. The formation of uniform compact grains in the microstructure of PbS films is achieved which is a consequence of multiple nucleation followed by one-step growth^47^.

Evaluating the morphology and composition of the obtained film showed that the PbS thin film deposited through dipping the substrates inside the chemical bath for 60 min have low surface roughness and full coverage of electron transport layer (ETL) layer. Thus, it is a good candidate for using as a precursor layer for preparing final lead halide perovskite film.

### Chemical conversion of PbS to PbI_2_ films

**Figure 2:**
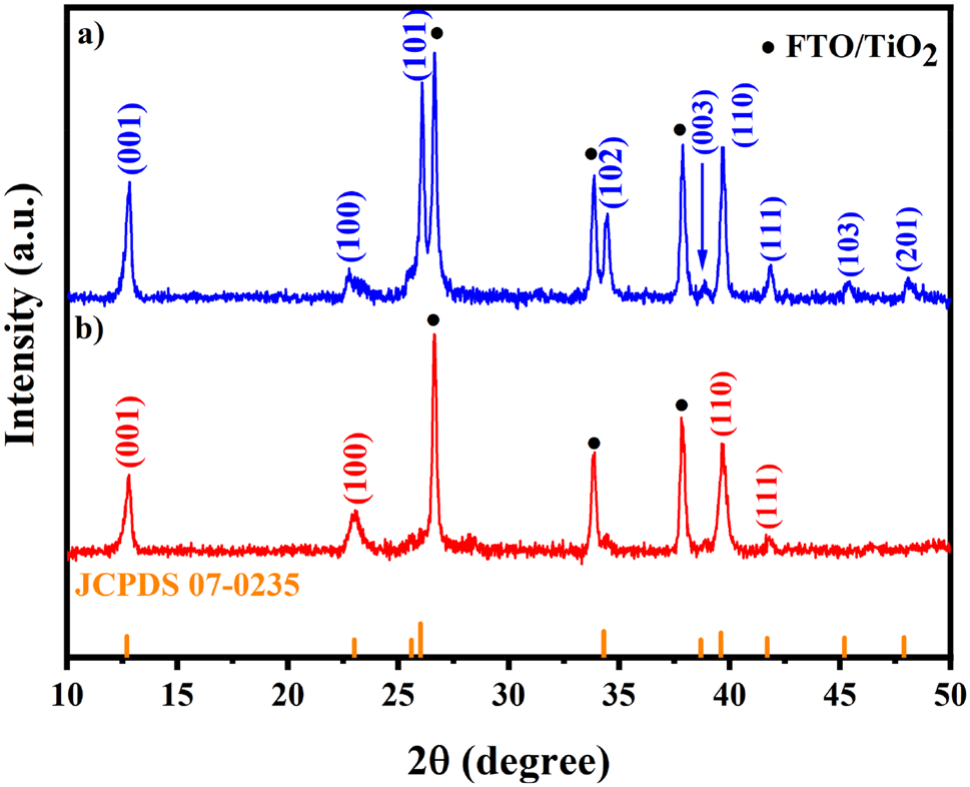
XRD patterns of PbI_2_ films obtained from iodination of chemically deposited PbS films at (**a**) 120 and (**b**) 155 °C.

The iodination of chemically deposited PbS films were carried out by putting PbS films in iodine vapor environment at two different temperatures of 120 and 155 °C. From XRD patterns (Fig. 2a,b), it is obvious that whole PbS is converted to PbI_2_ and instead of initial PbS peaks, some new peaks can be seen which are in accordance with crystallite planes of PbI_2_ (00–007-0235). The possible reaction can be^27^:

5 $${PbS+{I}_{2}}^{2+}\to Pb{I}_{2}+S\uparrow $$

The position of the main Bragg diffraction peaks are the same for two PbI_2_ films achieved at the two temperatures. However, there are some differences in the intensity of the peaks related to PbI_2_ crystallite planes, depending on the temperature of the reaction. The XRD pattern of 120 °C-PbI_2_ represented almost all the characteristic peaks of PbI_2_, including (001), (100), (101), (102), (003), (110), (111), (103) and (201) reflections. Whereas the pattern of 155 °C-PbI_2_ revealed four major peaks of (001), (100), (110) and (111). Analysis of the preferred orientation is performed through calculation of texture coefficients (TC). Table 1 shows the *TC(hkl)*s of the observed planes. For the 120 °C-PbI_2_, (001) plane has the highest *TC(hkl)* value. While for 155 °C-PbI_2_ the (100) plane is dominant. The change in crystallographic direction against the temperature of substrate is in agreement with results observed in vapor-deposited PbI_2_ crystals^48^. Provided that general conditions for producing diffraction peaks persist, i.e. Bragg’s law and parallel normal vector of planes to the diffraction vector^49^, we assume that in our experiments XRD only have detected diffractions from the crystallographic planes oriented parallel to the surface of the sample^50^. Thus, we expect that this variation of crystallographic texture observed here may be originated from the difference in microstructure features.

**Table 1:** The texture coefficients in for the crystal planes of PbI_2_ chemically bath deposited at different temperatures.

	Texture coefficient								
	(001)	(100)	(101)	(102)	(003)	(110)	(111)	(103)	(201)
120 °C-PbI_2_	2.42	0.62	0.79	0.80	0.89	1.53	1.10	0.52	0.34
155 °C-PbI_2_	0.78	2.39	–	–	–	0.58	0.25	–	–

**Figure 3:**
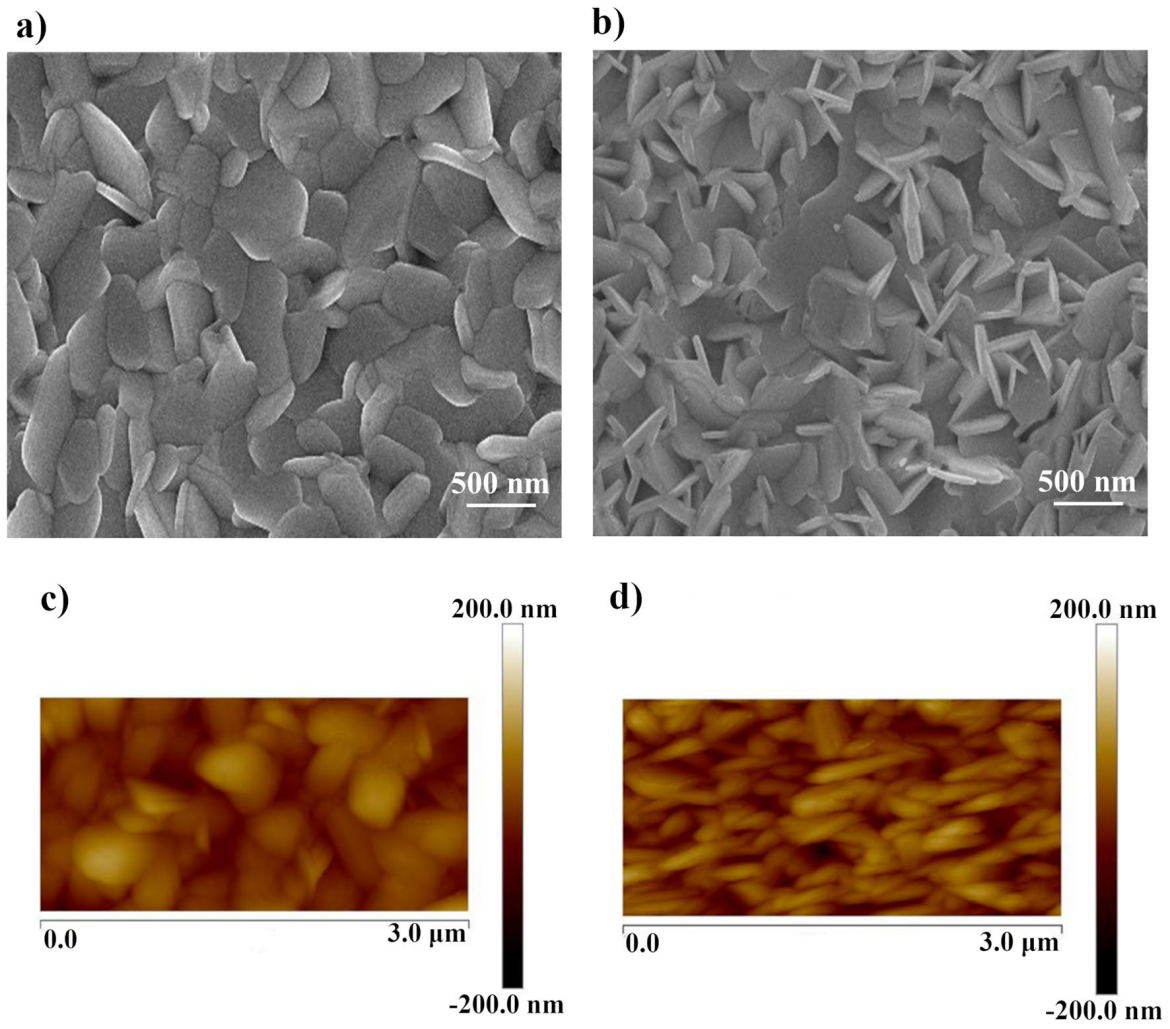
Top surface SEM images and AFM images of PbI_2_ films obtained from iodination of chemically deposited PbS films at (**a**, **c**) 120 and (**b**, **d**) 155 °C.

Figure 3a,b show the SEM micrographs of PbI_2_ films prepared at 120 and 155 °C, respectively. PbI_2_ layers fabricated by our present method exhibit clear grain boundaries and incompact structure, different from the PbI_2_ layers fabricated by conventional spin coating method which have fuzzy domain boundaries and few grain boundaries.

Furthermore, as expected from XRD results, there is an obvious difference amongst the structure of PbI_2_ layers obtained at two different temperatures. As can be seen from Fig. 3a, 120 °C-PbI_2_ has platelet crystals with hexagonal shape, which are lying parallel to substrate. This structure is consistent with the calculated TC coefficients. According to TC results shown in Table 1, the preferred orientation for 120 °C-PbI_2_ is (001) plane that is perpendicular to the *c-axis* of the hexagonal crystal system. Thus, the PbI_2_ is composed of a layered structure with basal planes parallel to the substrate. In contrast, in the case of 155 °C-PbI_2_, the highest TC is for the (100) plane which is parallel to the *c-axis* of the hexagonal crystal. Accordingly, as can be seen from Fig. 3b, many platelet-like grains are positioned perpendicular to the substrate. This peculiar orientation causes the formation of many intergranular voids which is the cause of the observed porous PbI_2_ layer. The AFM images of aforementioned PbI_2_ films are also taken and presented at Fig. 3c,d, respectively. The AFM images certifies the same morphology for achieved films.

### Production of final mixed-cation mixed halide perovskite films

It has been well demonstrated that changing the morphology of PbI_2_ film will provide a chance to modify the morphology and quality of perovskite layer, which may influence the PSC device performance^51^. In this regard, we have investigated the effect of two different PbI_2_ films on the solar cell properties.

**Figure 4:**
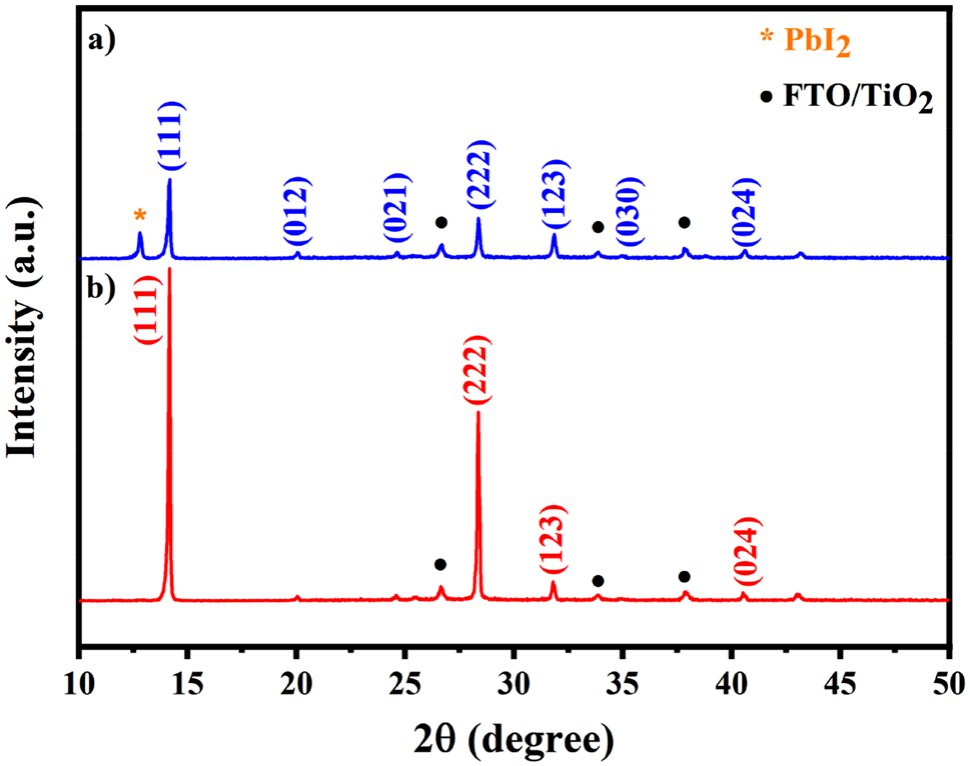
XRD patterns of perovskite films converted from (**a**) 120 °C-PbI_2_ and (**b**) 155 °C-PbI_2_.

Perovskite active layers were formed by spin coating of a solution of FAI:MACl:MABr (85:10:10 mg in 1 ml IPA) on top of as-converted PbI_2_ layers on mp-TiO_2_/bl-TiO_2_/FTO. As shown in Fig. 4a,b, the composition of the perovskite films achieved after iodination of 120 °C-PbI_2_ and 155 °C-PbI_2_ were characterized by XRD. After the third step, the presence of XRD peaks at 2θ = 14.16°, 20.04°, 24.6°, 28.36°, 31.8°, 34.92°, 40.52° and 43°, corresponding to the reflections from (111), (012), (021), (222), (123), (030), (024) and (333) planes of α-phase perovskite^52^, respectively, clarifies the formation of perovskite structure. Meanwhile, there is no trace of XRD peaks of δ-phase at 11.6°, indicating that selected composition and method is successful to achieve desirable α-phase perovskite. For perovskite film prepared from 120 °C-PbI_2_ an additional peak at 12.8° corresponding to (001) lattice plane of PbI_2_ suggests the incomplete conversion to perovskite. In contrast, in the case of perovskite from 155 °C-PbI_2_, the peaks of PbI_2_ completely disappear, indicating the complete conversion into photoactive black phase. In addition, for the former perovskite all the peaks are enhanced in intensity than those of the latter perovskite. It means that modifying the precursor PbI_2_ morphology may also increase the crystallinity of final perovskite film.

**Figure 5:**
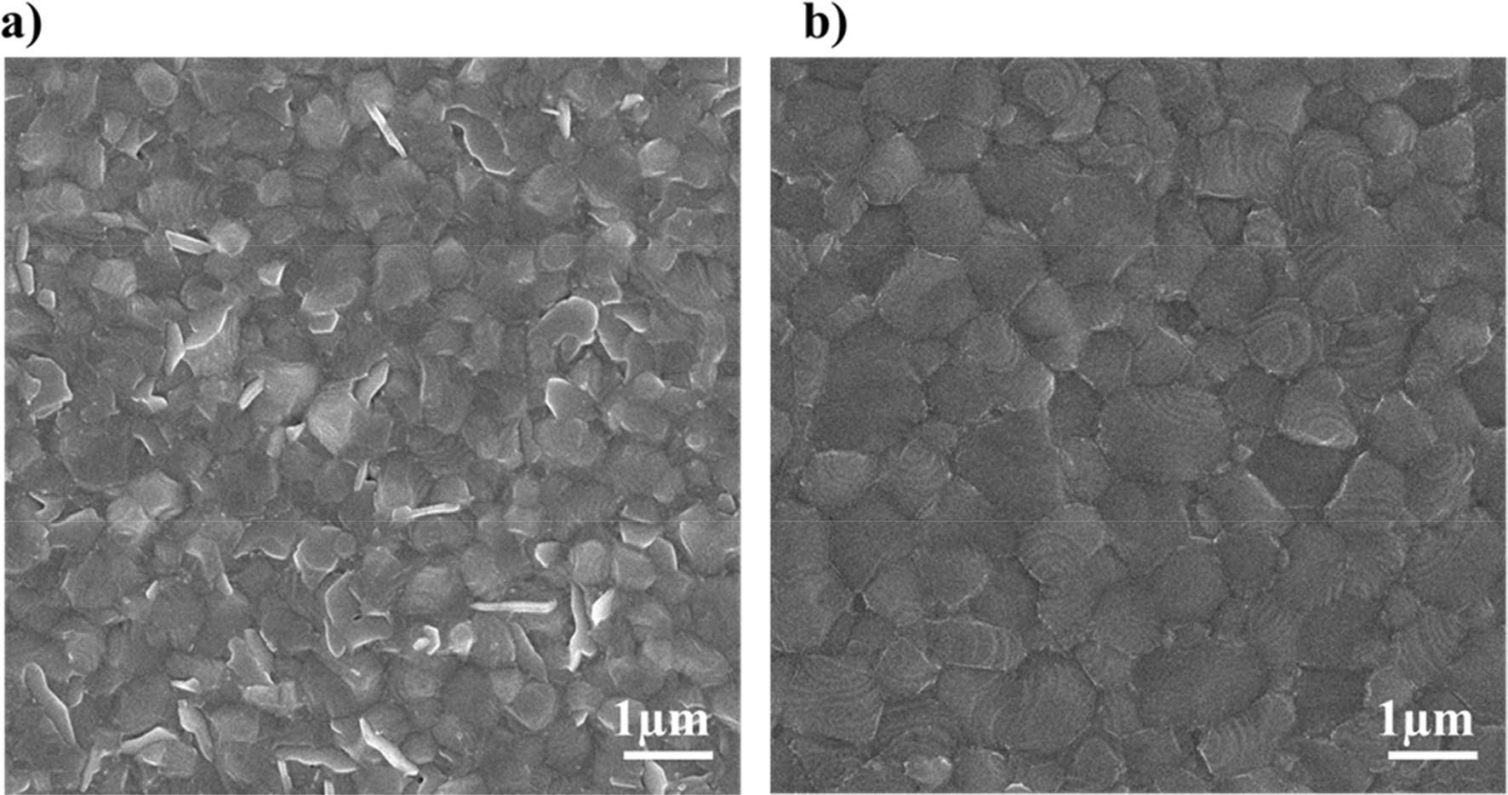
SEM images of perovskite films converted from (**a**) 120 °C-PbI_2_ and (**b**) 155 °C-PbI_2_.

The SEM images taken from the top surface of the final perovskite films, made from PbI_2_ layer converted at two temperatures of 120 and 155 °C, are shown in Fig. 5a,b, respectively. The perovskite film produced from the 120 °C-PbI_2_ layer exhibits the existence of disconnected grains with edges which may be unconverted PbI_2_ since heavy-atom regions appear with brighter contrast in the SEM images^53^. EDX analysis of the white regions also confirms the speculated composition, (Fig. S1). In contrast, the perovskite film produced from the 155 °C-PbI_2_ layer shows smooth and homogenous perovskite grains with size up to 800 nm which is comparable to that observed for perovskite films prepared by a conventional spin-coating method^54^. Furthermore, some spiral-shape lines can be seen on grains. According to the theory of crystal growth, it can be the result of a spiral growth mechanism, which leads to a step-like morphology^55^.

According to both XRD and SEM results, it can be concluded that the reaction rate of 155 °C-PbI_2_ is faster than that of 120 °C-PbI_2_. In the case of 155 °C-PbI_2_, the vertically arranged nanoplatlets create a large degree of porosity on the surface of PbI_2_ film. These pores facilitate the permeation of FAI:MACl:MABr solution in the whole pbI_2_ layer, which in turn would favor the full conversion of PbI_2_ to final perovskite material. Fast conversion is of great importance in the sequential deposition process because residual PbI_2_ can act as an insulate layer.

**Figure 6:**
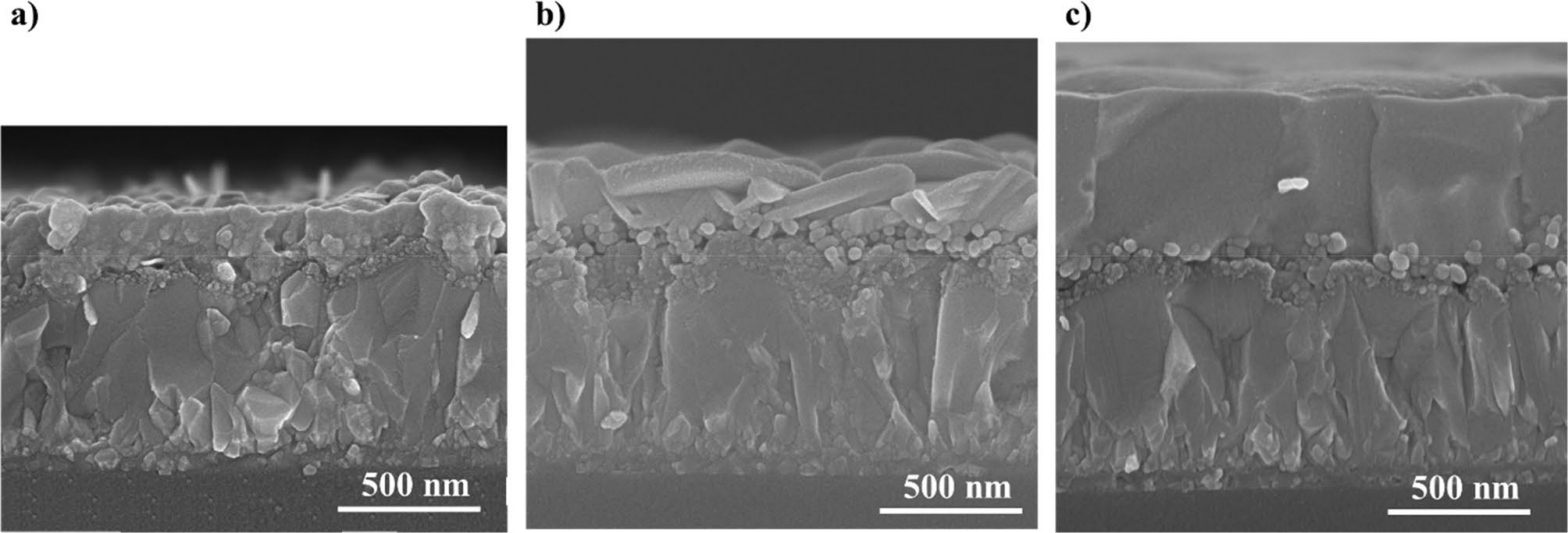
Cross sectional SEM images of (**a**) chemically deposited PbS, (**b**) 155 °C-PbI_2_ and (**c**) FAPbI_3_-based film produced from 155 °C-PbI_2_ on mp-TiO_2_/bl-TiO_2_/FTO substrate.

Cross sectional SEM images of chemically deposited PbS, 155 °C-PbI_2_ and FAPbI_3_-based film produced from 155 °C-PbI_2_ on mp-TiO_2_/bl-TiO_2_/FTO substrate are provided in Fig. 6a–c, respectively. Comparing Fig. 6a,b, it can be seen that the thickness value of PbI_2_ is almost twice of PbS. According to Fig. 6c the thickness of FAPbI_3_-based perovskite after converting PbI_2_ increased by a factor of 2 again. We interpret the thickening of the deposited films by the volume expansion happening after chemical conversion at each step^20,26^. The volume expansion is attributed to variation of lattice parameters of PbS, PbI_2_ and perovskite.

**Figure 7:**
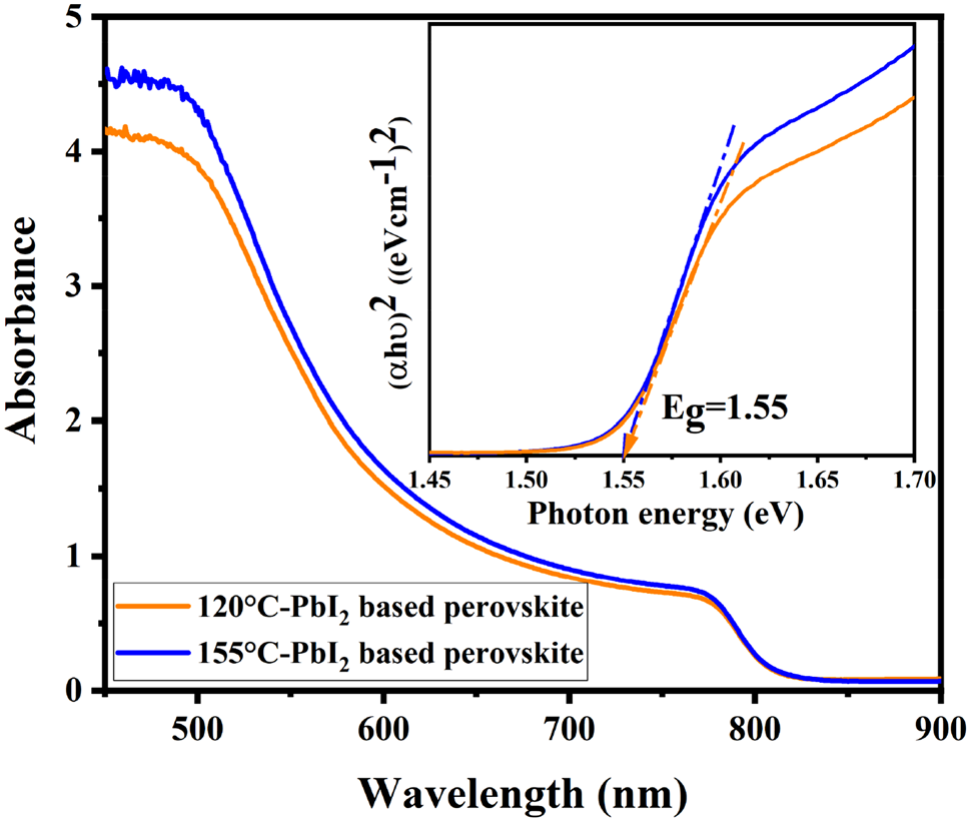
Absorption spectrum for the FAPbI_3_-based films produced from 120 °C-PbI_2_ to 155 °C-PbI_2_. Inset shows the Tauc plots to estimate the bandgap of the films.

Figure 7 shows the UV absorption spectra of two samples prepared from two different PbI_2_ layers. The higher absorption of perovskite film based on 155 °C-PbI_2_ compared to perovskite film based on 120 °C-PbI_2_ may be related to the higher amount of perovskite in the former one. Both spectra show onsets of absorption at 810 nm. The optical band gap almost remains the same (1.55 eV) for both films. A range of E_g_ values between 1.51 and 1.55 eV have been reported for (FA/MA)Pb(I/Br) mixed-cation mixed halide perovskites^56–62^ which is consistent with our present result. Therefore, tuning the composition of perovskite by partially substituting cation and halide resulted in an increase in the bandgap compared to pure FAPbI_3_ (1.45–1.51 eV in thin films)^54^. Widening of the band gap can be correlated with the perturbing the organic cation size that can cause the contracting of the whole lattice and the change of B–X bond length, which has been shown to influence E_g_ in ABX_3_ structure^63^.

UV absorption spectra also confirm the formation of α-phase of FAPbI_3_ without the presence of any trace of δ-phase. Because the yellow phase of FAPbI_3_ has characteristic absorption peak below 500 nm^64^, whereas both the obtained spectra have shown a remarkable absorption in the visible range. Obtaining pure α-phase of FAPbI_3_ is of great importance for photovoltaic applications. The yellow δ‐FAPbI_3_ is a non-photoactive phase and due to having a chain‐like structure prevents the transport of electrons and in turn reduces photovoltaic performance^65^.

### PSC fabrication and device performance

**Figure 8:**
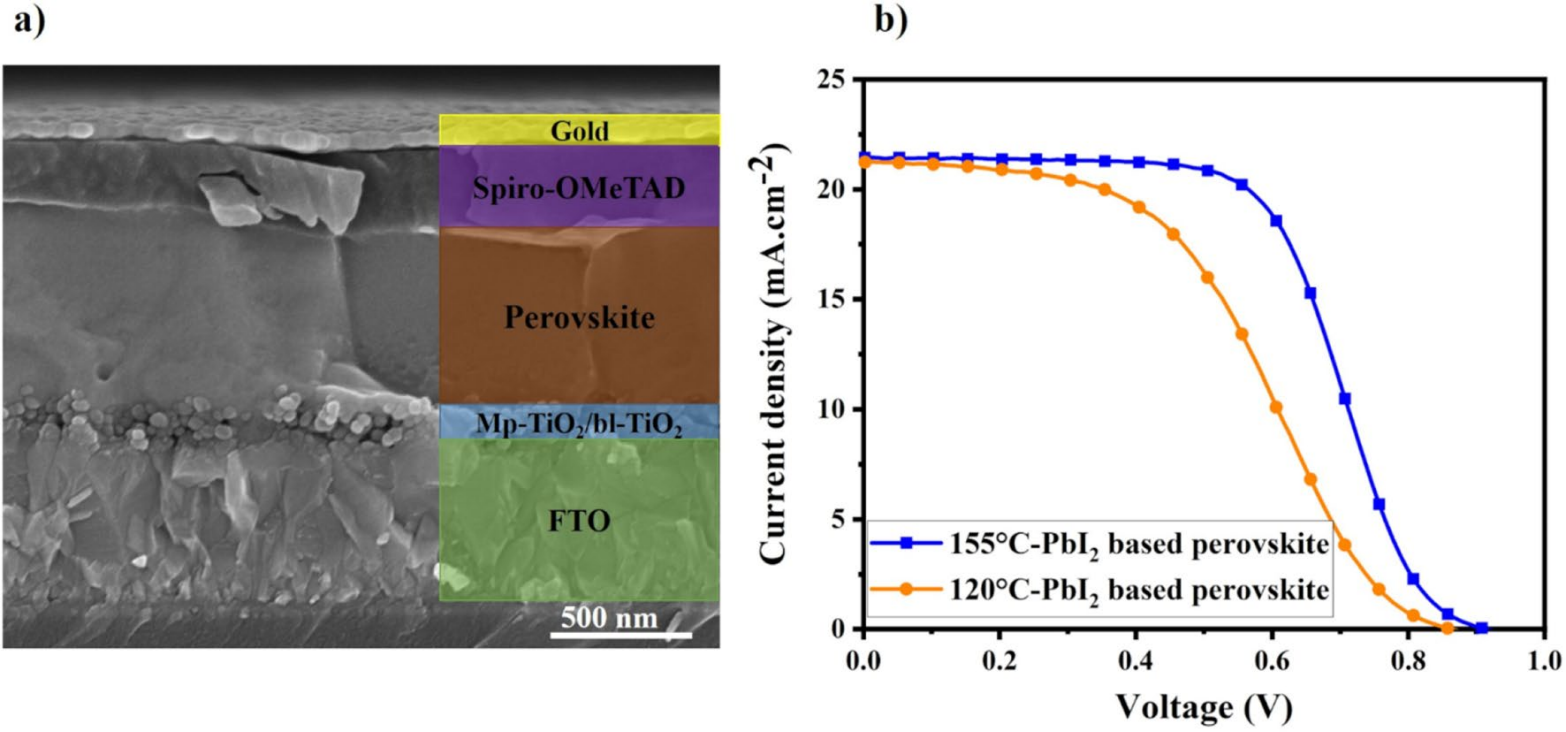
(**a**) Cross section SEM image of a FTO/bl-TiO_2_/mp-TiO_2_/perovskite/Spiro-OMeTAD/Au solar cell. (**b**) Current–voltage characteristic of the best-performance devices based on 120 °C-PbI_2_ and 155 °C-PbI_2_.

In order to investigate the photovoltaic applicability of the synthesized perovskite films, devices with configuration FTO/bl-TiO_2_/mp-TiO_2_/perovskite/spiro-OMeTAD/Au were made by using perovskite films obtained from three-step-method. Figure 8a shows the cross sectional view of the complete perovskite cell and the right part of image was colored and labeled to make a guide to position and thickness of each layer. It can be seen that perovskite film prepared by multi-step method has columnar crystal grains which are elongated from bottom to top.

The related current density–voltage (J–V) curves of the best perovskite fabricated from perovskite based on 120 °C-PbI_2_ and 155 °C-PbI_2_ measured under AM 1.5G solar illumination at 100 mW cm^−2^ and are plotted in Fig. 8b. Also Table 2 summarizes the photovoltaic parameters obtained from J–V curves, average values (based on six devices) and corresponding standard deviation. Tables S3 and S4 in the “Supplementary document S1” shows photovoltaic parameters and error analysis derived from J–V curves of devices. Furthermore, the corresponding box chart plots of J_sc_, V_oc_, FF and PCE for two different processing conditions can be found in Fig. S2a–d, respectively. As can be seen from Table 2, the best-performing cell fabricated via the proposed method, demonstrates acceptable performance with a *J*_*sc*_ of 21.43 mA cm^−2^, V_oc_ of 0.918 V, FF of 57.67 and PCE of 11.35%. It can be seen that the J_sc_ of PSC based on 155 °C-PbI_2_ increased slightly compared to that of 120 °C-PbI_2_ based perovskite. It can be related to PbI_2_-free and high crystalline perovskite synthesized from 155 °C-PbI_2_ which leads to increment of visible light absorption. Furthermore, in the device based on 120 °C-PbI_2_, the leftover PbI_2_ can hinder the electron transfer of perovskite film due to high resistivity of PbI_2_ which in turn increases the possibility of charge carrier recombination and reduces *V*_*oc*_ and *FF*.

**Table 2:** Photovoltaic parameters of PSCs fabricated by chemical bath deposition-based method.

	*V*_*oc*_ (V)	*J*_*sc*_ (mA cm^−2^)	*FF* (%)	*PCE* (%)
120 °C-PbI_2_ based	0.857 (0.845 ± 0.03)	21.24 (21.02 ± 0.33)	45.04 (43.98 ± 1.03)	8.20 (7.82 ± 0.34)
155 °C-PbI_2_ based	0.918 (0.904 ± 0.01)	21.43 (21.47 ± 0.10)	57.67 (57.06 ± 0.89)	11.35 (11.08 ± 0.32)

The values in parentheses correspond to the average values from six devices and the corresponding standard deviation.

Totally, the performance of the device prepared by the proposed three-step method is comparable with the performance of other devices made from uncommon metal precursors rather than PbI_2_ and is the highest reported efficiency for CBD-based method of synthesizing perovskite. Nevertheless, the method is still less efficient compared to the conventional spin coating method and there are many rooms to modify different parameters of this method in order to enhance the efficiency. One of the main reasons for low efficiency might be the appearance of so- called S-shaped J–V curve, as is evident in Fig. 8b. This phenomenon is one of the many problems occurring when developing new materials and new methods for fabricating perovskite solar cell devices. In most cases, this observation is attributed to the presence of extraction barriers and large interface resistance^66^, which can lead to the accumulation of photo-generated charges and a charge reservoir. Whereas the occurrence of these interface barriers might have different origins, which needs more detailed and systematic study.

## Conclusion

Briefly, a scalable processing technique with environmental-friendly approach by neglecting DMF from conventional fabrication processes of halide perovskite solar cells was successfully developed by using chemically deposited PbS from an aqueous solution as the precursor and created perovskite films with good quality and full coverage. The iodination temperature in the second step was changed in a controlled way and the influence on final film quality was evaluated. Finally, grown perovskite films with no unconverted PbI_2_ was synthesized by iodination of the PbS layer at 155 °C and exposing to the FAI:MACl:MABr/IPA solution. This was subsequently applied in solar cell devices with the following structure: FTO/bl-TiO_2_/mp-TiO_2_/perovskite/spiro-OMeTAD/Au. The assembled solar cells represented the *PCE* of 11.35% with corresponding photocurrent of 21.43 mA cm^−2^ and open circuit voltage of 0.918 V. CBD is a widely used industrial method, therefore, the offered technique opens new avenues for large-scale production of perovskite solar cells. Moreover, avoiding the use of toxic solvent especially DMF facilitates the green fabrication of PSCs. It can be expected that the performance of devices made by this method will be enhanced via optimizing the various parameters of the deposition.

## Materials and method

### Preparation of electron transport layers

Fluorine-doped tin oxide (FTO) substrates (Pilkington, TEC8, 8 Ω cm^−2^) were undergone surface preparation including pattern etching and ultrasonically cleaning in acetone, detergent and ethanol for 30 min per each. A dense TiO_2_ blocking layer (bl-TiO_2_) was deposited on the FTO via the spray pyrolysis method from a solution of titanium diisopropoxide bis(acetylacetonate) (Aldrich) diluted in ethanol (v/v, 1/10) at 450 °C. Then, a 200-nm thick mesoporous TiO_2_ (mp-TiO_2_) layer was fabricated on the substrates by spin coating a TiO_2_ commercial paste (containing TiO_2_ nanoparticles: average diameter: 50 nm, anatase) diluted in 2-methoxyethanol and terpineol (Aldrich) solution followed by annealing at 500 °C for 60 min and cooling to room temperature.

### Synthesizing of the perovskite film

A three-step deposition method was employed to produce the perovskite film. Initially a PbS layer was coated on substrates via chemical bath deposition method. The deposition bath contained 85 ml of an aqueous solution, consisting of 2.5 ml Pb(CH_3_COO)_2_·3H_2_O (Sigma-Aldrich) 1 M, 6 ml thiourea SC(NH_2_)_2_ (Sigma-Aldrich) 1 M, 10 ml NaOH (Aldrich) 1 M and 2 ml triethanolamine (Sigma) 1 M. The deposition was performed at room temperature. Afterward, the PbS layers and iodine chips (Sigma-Aldrich) were put inside one petri dish at different temperatures to chemically convert PbS to PbI_2_. The temperatures were chosen according to iodine phase diagram to obtain the iodine vapor. In fact, it is a known that iodine can be in vapor state at the atmospheric pressure in a range of 113.5–184.4 °C. In this range, the iodine liquefies first and then transforms to violet-colored gas. Therefore, two different temperatures in this range were chosen to simultaneously sublimate the iodine and react with the PbS. Next, PbI_2_ films were exposed to a FAI:MACl:MABr solution (85:10:10 mg ml^−1^) and then spun at 5000 rpm for 30 s. The selection of solution was based on a comparison between the qualities of perovskite films obtained by different solutions as shown in Fig. S3. After two step annealing at 150 °C and 100 °C for 30 min per each, the conversion of PbI_2_ to perovskite film was completed.

### Assembling perovskite solar cells (PSCs)

After preparing FAPbI_3_-based perovskite/mp-TiO_2_ /bl-TiO_2_, a 2,20, 7,70-tetrakis (N,N-di-pmethoxyphenylamino)-9,90-spirobifluorene (spiro-OMeTAD, Lumtec) processor solution (100 mg of spiro-OMeTAD, 39 ml of 4-tert-butylpyridine (Aldrich), 23 ml of bis (trifluoromethane) sulfonamide lithium salt (Li-TFSI, Aldrich) solution and 10 ml of cobalt salt (Lumtec) solution per 1.1 ml chlorobenzene) was spin coated onto the as-deposited perovskite film described above at 3000 rpm for 30 s. Finally, for completing the device an 80 nm gold film was coated on top of layers through thermal evaporation method.

### Characterization

The thickness of the deposited and chemically converted solid state thin films were measured by a stylus profiler (KLA Tencor). The morphologies of the films were examined using a scanning electron microscopy (SEM, S-4800, Hitach High-Technologies) and atomic force microscopy (AFM, MultiMode V Veeco). Energy dispersive X-ray analysis (EDX) is done in conjunction with SEM. Crystalline structure of the films were determined by using a X-ray diffractometer (XRD, D8 ADVANCE, Bruker AXS) with Cu Kα radiation (*λ* = 1.5405 Å).

In order to estimate the degree of crystalline orientation in different directions, the texture coefficient (*TC*) was calculated by Eq. (6)^48^:

6 $$ TC(hkl) = \frac{{I(hkl)/I_{0} (hkl)}}{{{{\left[ {\sum {I(hkl)/I_{0} (hkl)} } \right]} \mathord{\left/ {\vphantom {{\left[ {\sum {I(hkl)/I_{0} (hkl)} } \right]} N}} \right. \kern-\nulldelimiterspace} N}}} $$

where *I*(*hkl*) is the intensity of the measured peak, *I*_*0*_(*hkl*) is the intensity of reference data peak for random oriented powder, and N is the number of measured peaks.

The absorption spectrum of final perovskite layers was extracted using a UV–Vis spectrophotometer (Jasco V-780). In order to record current–voltage (J–V) graphs, A Keithley 2400 source meter was used and Illumination of air mass (AM) 1.5 G and a power of 100 mW cm^−2^ was applied by a solar simulator (Newport, Oriel Class A, 91195A). For setting the correct irradiance of the solar simulator, a calibrated Si reference cell certified by National Renewable Energy Laboratory (NREL) was used. For each condition, the photovoltaic parameters with corresponding error analysis extracted from J–V curves of all devices.

## Supplementary Information

Supplementary Information.
