# Supplementary material for: Towards environmental friendly multi-step processing of efficient mixed-cation mixed halide perovskite solar cells from chemically bath deposited lead sulphide
Source: Sci Rep. 2021 Sep 17;11:18561. doi: 10.1038/s41598-021-97633-5 (PMC8448853; doi:10.1038/s41598-021-97633-5)
Supplement: Supplementary file 1 — Supplementary Information. [file 41598_2021_97633_MOESM1_ESM.docx]

Supplementary information

**Towards environmental friendly multi-step processing of efficient mixed-cation mixed halide perovskite solar cells from chemically bath deposited lead sulphide**

Sahel Gozalzadeh^1^, Farzad Nasirpouri^1,*^, Sang Il Seok^2^

^1^*Faculty of Materials Engineering, Sahand University of Technology, Tabriz 51335-1996, Iran*

^2^*School of Energy and Chemical Engineering, Ulsan National Institute of Science and Technology (UNIST), 50 UNIST-gil, Eonyang-eup, Ulju-gun, Ulsan 689-798, Republic of Korea*

*-Corresponding author: [nasirpouri@sut.ac.ir](mailto:nasirpouri@sut.ac.ir); [f_nasirpouri@yahoo.com](mailto:f_nasirpouri@yahoo.com)

**Table S1.** Representative of different water-based metal precursors for synthesizing perovskite film and fabricating PSC.

| Metal precursor | Processing route | PSC configuration | PCE(%) | Year/Ref |
| --- | --- | --- | --- | --- |
| Pb | Magnetron sputtering of Pb film  Spin coating of MAI/IPA solution | FTO/ZnO/MAPbI_3_/spiro-OMeTAD/Au | 3.08 | 2016 ^16^ |
| PbO | Electrodeposition of PbO  Exposing in iodine vapors  Spin coating MAI | FTO/mp-TiO_2_/MAPbI_3_/spiro-OMeTAD/Au | 12.5 | 2015 ^17^ |
| PbO | Electrodeposition of PbO film  Spin-coating a layer of MAI | FTO/mp-TiO_2_/MAPbI_3_/spiro-OMeTAD/Au | 14.59 | 2015 ^18^ |
| PbO_2_ | Anodic ED of PbO_2_  Exposing PbO_2_ to an excess of HI vapor  Exposing PbI_2_ to MAI vapor | - | - | 2016 ^19^ |
| PbO_2_ | Electrodeposition of PbO_2_  Immersing in HI/ethanol solution  Immersing PbI_2_ samples in MAI solution | FTO/bl-TiO_2_/mp-TiO_2_/MAPbI_3_/carbon electrode | 10.19 | 2015 ^20^ |
| Pb(NO_3_)_2_ | Spin coating  Spin coating MAI solution | FTO/mp-TiO_2_/MAPbI_3_/spiro-OMeTAD/Au | 13.7 | 2017 ^21^ |
| Pb(NO_3_)_2_ | Spin coating  Dipping in MAI solution | FTO/mp-TiO_2_/MAPbI_3_/spiro-OMeTAD/Ag | 13.7 | 2017 ^22^ |
| Pb(NO_3_)_2_ | Spin coating  Dipping in MAI solution | FTO/bl-TiO_2_/mp-TiO_2_/MAPbI_3_/spiro-OMeTAD/Au | 12.58 | 2015 ^23^ |
| Pb(NO_3_)_2_ | Sequential dipping in Pb(NO_3_)_2_ and MAI solution | FTO/bl-TiO_2_/mp-TiO_2_/ZnO/ MAPbI_3_/spiro-OMeTAD/MoO_3_/Ag | 12.41 | 2018 ^24^ |
| PbSe | Physical vapor deposition of PbSe  Exposing to iodine vapor  In situ closed-space vapor transport for conversion to MAPbI_3_ | - | - | 2019 ^25^ |
| PbS | Atomic layer deposition of PbS  Exposure to iodine gas  Dipping in (MAI) solution | - | - | 2015 ^26^ |
| PbS | Depositing PbS via rf-sputtering  Exposure to iodide gas (I_2_)  Dipping into MAI solution | - | - | 2018 ^27^ |
| PbS | Chemical bath deposition of PbS  Transforming to CH_3_NH_3_PbI_3_ by CVD process | FTO/C-TiO_2_/CH_3_NH_3_PbI_3_/Spiro-MeOTAD/Ag | 4.65 | 2016 ^28^ |
| PbS | Electrodeposition of PbS  Exposure to iodide gas (I_2_)  Dipping into MAI solution | FTO/bl-TiO_2_/mp-TiO_2_/MAPbI_3_/spiro-OMeTAD/Au | 7.72 | 2021 ^29^ |

**Table S2.** Thickness of PbS, PbI_2_ and perovskite layer versus immersion duration inside chemical bath.

| Immersion time | Thickness (nm) | | |
| --- | --- | --- | --- |
|  | PbS | PbI_2_ | perovskite |
| 30 | 60 | 130 | 320 |
| 60 | 158 | 310 | 600 |
| 90 | 200 | 400 | 800 |


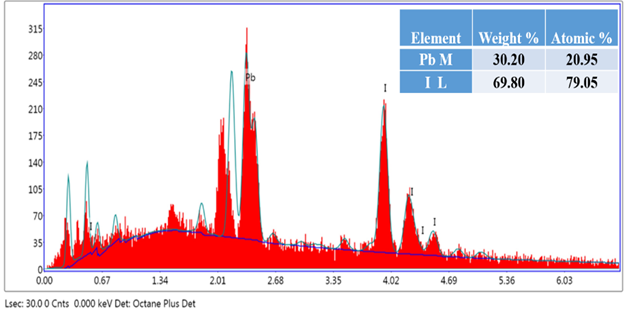


**Figure S1**. Energy dispersive X-ray analysis (EDAX) of bright regions in perovskite structure. Table in the inset of Figure shows the atomic percentage of the constituent elements present in the sample.

**Table S3.** Photovoltaic parameters of FTO/bl-TiO_2_/mp-TiO_2_/FA-based perovskite /spiro-OMeTAD/Au solar cells chemically deposited PbS as the metal precursor by using three step method (i) chemical bath deposition of PbS ii)iodination at 120°C iii) exposing to FAI/MACl/MABr solution)

|  | *V_oc_* | *J_sc_* | *FF* | *PCE* |
| --- | --- | --- | --- | --- |
|  | 0.857 | 21.24 | 45.04 | 8.20 |
|  | 0.837 | 20.49 | 42.43 | 7.28 |
|  | 0.867 | 21.25 | 43.15 | 7.96 |
|  | 0.837 | 20.76 | 44.45 | 7.73 |
|  | 0.797 | 21.32 | 44.92 | 7.64 |
|  | 0.877 | 21.06 | 43.87 | 8.11 |
|  |  |  |  |  |
| avg. | 0.845333 | 21.02 | 43.97667 | 7.82 |
| stdev. | 0.028577 | 0.329059 | 1.032621 | 0.340646 |
|  |  |  |  |  |

**Table S4.** Photovoltaic parameters of FTO/bl-TiO_2_/mp-TiO_2_/FA-based perovskite /spiro-OMeTAD/Au solar cells chemically deposited PbS as the metal precursor by using three step method (i) chemical bath deposition of PbS ii)iodination at 155°C iii) exposing to FAI/MACl/MABr solution)

|  | *V_oc_* | *J_sc_* | *FF* | *PCE* |
| --- | --- | --- | --- | --- |
|  | 0.918 | 21.43 | 57.67 | 11.35 |
|  | 0.908 | 21.42 | 58.02 | 11.28 |
|  | 0.877 | 21.44 | 55.67 | 10.48 |
|  | 0.898 | 21.58 | 56.72 | 10.99 |
|  | 0.908 | 21.37 | 57.71 | 11.20 |
|  | 0.918 | 21.61 | 56.57 | 11.23 |
|  |  |  |  |  |
| avg. | 0.9045 | 21.475 | 57.06 | 11.08833 |
| stdev. | 0.015411 | 0.096488 | 0.894874 | 0.321709 |


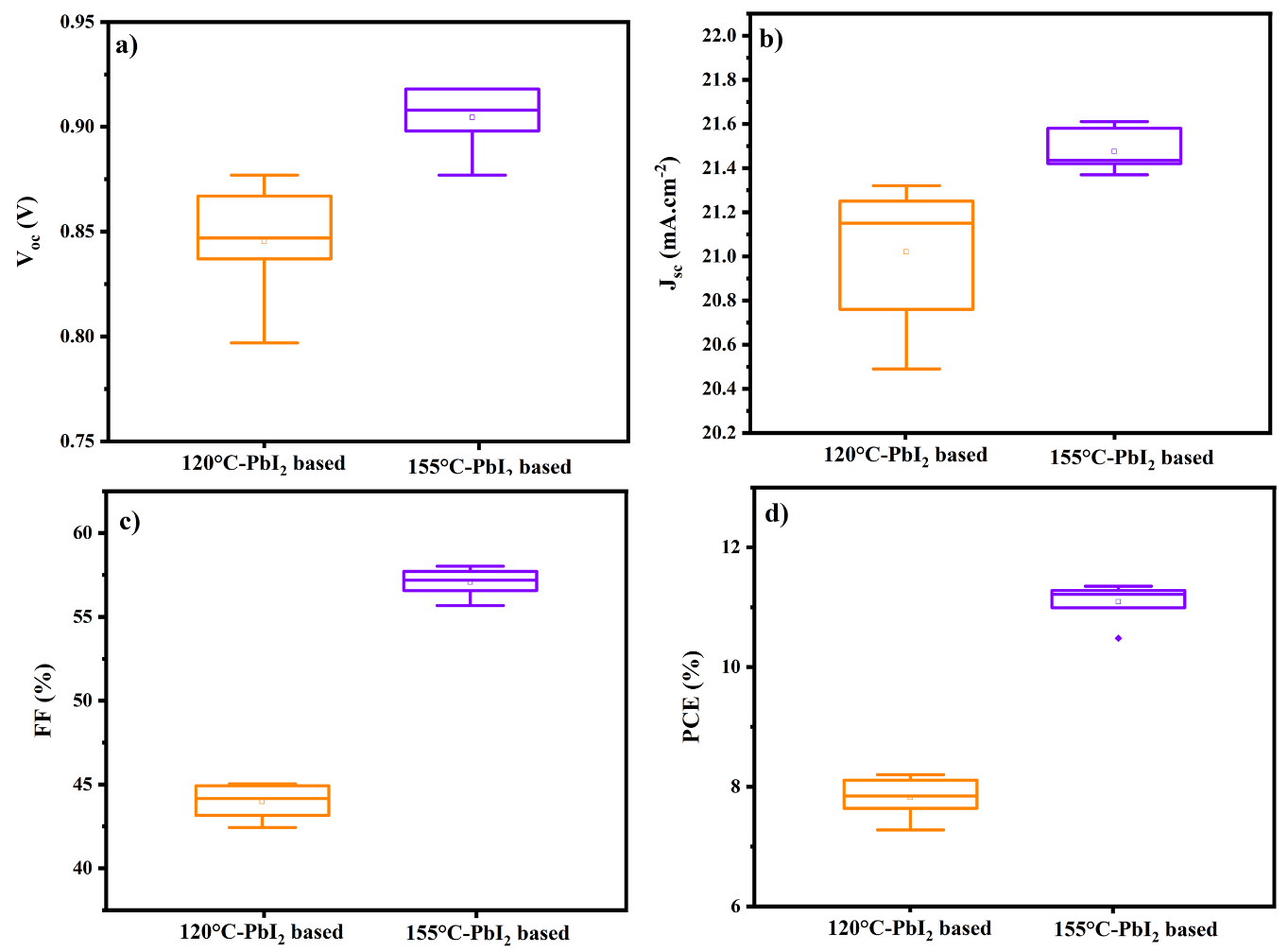


**Figure S2**. Statistical histograms of a)V_oc_, b) J_sc_, c) FF and d) PCE for devices based on perovskite films converted from 120°C-PbI_2_ and 155°C-PbI_2_. Every box shows the parameter distribution of 6 devices.

**
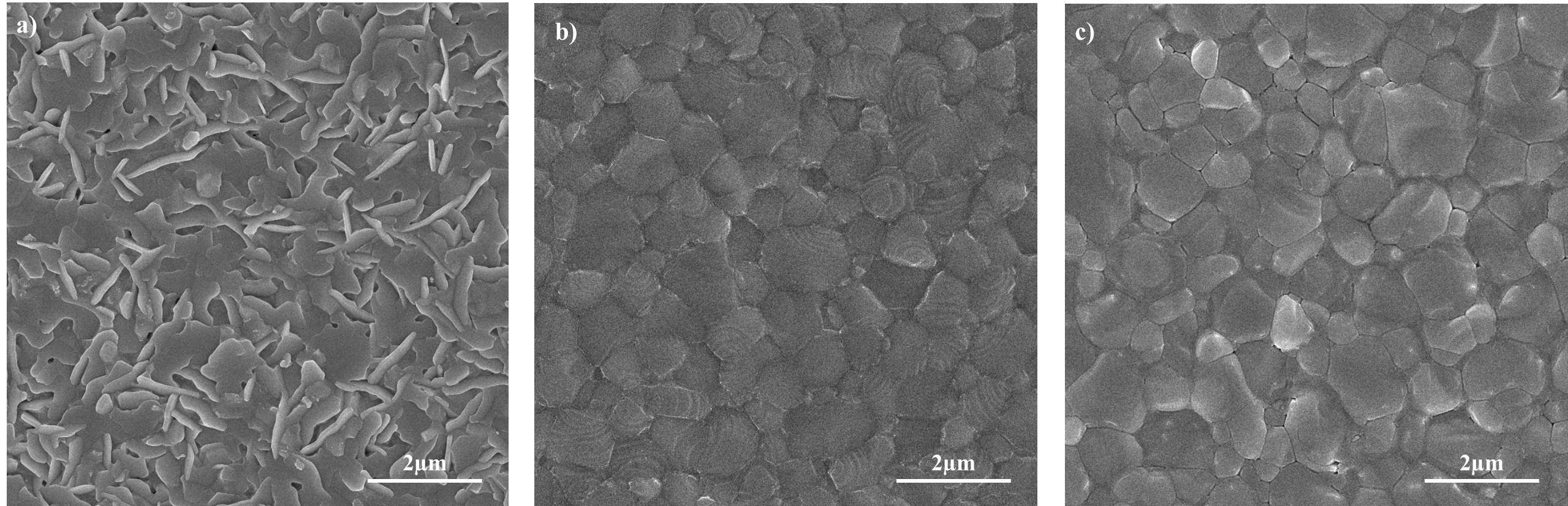
**

**Figure S3**. SEM images of perovskite films obtained from spin coating of three different FAI:MACl:MABr solutions including a)70:10:10 mg in 1 ml IPA b)85:10:10 mg in 1 ml IPA and c)100:10:10 mg in 1 ml IPA on PbI_2_.
